# ‘Triple clear’: a systematic and comprehensive surgical process for Campanacci grades II and III giant cell tumors of the bone, with or without pathological fracture and slight joint invasion

**DOI:** 10.1186/s12957-023-02982-2

**Published:** 2023-03-29

**Authors:** Yushan Wang, Pengfei Shao, Qiaoqiao Tian, Haoze Li, Jian Li, Peng Ren, Zhi Lv, Jia Lv, Junjun Bai, Yi Feng

**Affiliations:** Department of Orthopaedics, The Second Hospital of Shanxi Medical University, No. 382 of 51 Road, Taiyuan, Shanxi China

## Abstract

**Background:**

In recent years, researchers have proposed a number of adjuvant methods for extended curettage of giant cell tumors of the bone. However, various schemes have significant differences in efficacy and safety. Therefore, this article will describe an empirical expanded curettage protocol, ‘triple clear’, in detail to show the effect of the efficient surgical protocol.

**Method:**

Patients with Campanacci grades II and III primary GCTB who were treated with either SR (*n* = 39) or TC (*n* = 41) were included. Various perioperative clinical indicators, including the therapy modality, operation time, Campanacci grade, and filling material were recorded and compared. The pain level was determined by the visual analog scale. Limb function was determined by the Musculoskeletal Tumour Society (MSTS) score. Follow-up time, recurrence rates, reoperation rates, and complication rates were also recorded and compared.

**Result:**

The operation time was 135.7 ± 38.4 min in the TC group and 174.2 ± 43.0 min in the SR group (*P* < 0.05). The recurrence rates were 7.3% in the TC group and 8.3% in the SR group (*P* = 0.37). The MSTS scores at three months after surgery were 19.8 ± 1.5 in the TC group and 18.8 ± 1.3 in the SR group. The MSTS scores at two years were 26.2 ± 1.2 in the TC group and 24.3 ± 1.4 in the SR group (*P* < 0.05).

**Conclusion:**

TC is recommended for patients with Campanacci grade II–III GCTB and for those with a pathological fracture or slight joint invasion. Bone grafts may be more suitable than bone cement in the long term.

**Supplementary Information:**

The online version contains supplementary material available at 10.1186/s12957-023-02982-2.

## Synopsis

This article introduced a systematic and comprehensive surgical procedure (‘triple clear’) for patients with giant cell tumor of the bone. By reviewing the data, the clinical effects of segmental resection and ‘triple clear’ (TC) were compared. The effects of two implant materials, including allogeneic bone and bone cement, were also investigated. Finally, we concluded that TC should be the first choice of treatment for patients with GCTB and that bone grafts might provide more benefits than bone cement in the long term.

## Background

Giant cell tumor of the bone (GCTB) is a common bone tumor. However, compared with general benign tumors, GCTB is an osteolytic tumor with an underlying malignancy. The commonly used treatments for GCTB are curettage combined with adjuvant therapy and segmental resection (SR) with prosthesis reconstruction. Considering that most affected patients are between 20 and 40 years of age, curettage combined with adjuvant therapy is a more acceptable approach. Therefore, effective adjuvant therapy has become a focus of GCTB research. High-speed burring has been used to efficiently remove tumor tissues. Denosumab, a humanized monoclonal antibody that protects against the receptor activator of nuclear factor-κB-ligand (RANKL), has been approved for the treatment of advanced GCTB. However, it is not routinely used because of reports that show negative effects [1, 2]. A variety of inactivation methods have also been reported, including phenol [3], liquid nitrogen [4], hydrogen peroxide [5], ethanol [6], electrocauterization [7], argon beam coagulation [8] and hypertonic saline. Even though many inactivation methods have been investigated, the most appropriate method has not yet been determined. In past studies, researchers have compared various inactivation methods and concluded that various inactivation methods would produce different clinical results due to different methods of use. The relapse rate was not very different, so it was recommended that the safety of inactivation be the primary consideration [9, 10, 11, 12, 13, 14, 15, 16, 17, 18, 19, 20, 21, 22, 23, 24]. According to the body of relevant literature [9, 10, 11, 12, 13, 14, 15, 16, 17, 18, 19, 20, 21, 22, 23, 24] (Table 1), we have summarized a few key principles. (1) Careful scraping of the wall in the residual cavity was essential after tumor curettage. (2) Electrotome thermocoagulation (ET) and anhydrous ethanol (AE) infusion achieved low recurrence rates without adverse effects, which are ideal inactivation methods. However, no researchers have attempted to combine these two methods for the treatment of patients with GCTB. In this study, we propose the combination of AE infusion and ET. And we also present a systematic and efficient surgical procedure that may achieve better clinical outcomes.

**Table 1:** Safety comparison of different inactivation methods

**Method**	**Recurrence rate**	**Side effect**	**Safety**
**Phenol**	**11.5%-35.3%**	**Absorptive toxicity**	
		**Carcinogenicity**	**Medium**
		**Damage to vessels and nerves**	
		**Joint complications**	
**Absolute ethanol**	**9.5-11%**	**/**	**High**
**Electric knife**	**5.1%-33.3%**	**/**	**High**
ABC*	**16.6%**	**Postoperative fracture**	
		**Physeal arrests**	**Medium**
		**Synovitis**	
		**Bursitis**	
**Liquid nitrogen**	**7.5%-38%**	**Postoperative fracture**	
		**Skin necrosis**	
		**Infection**	
		**Transient nerve palsy**	**Low**
		**Traumatic arthritis**	
		**Bone graft nonunion**	
		**Cryo-shock syndrome**	

*ABC Argon Beam Coagulation

## Materials and method

### General patient characteristics and selection criteria

**Fig. 1:**
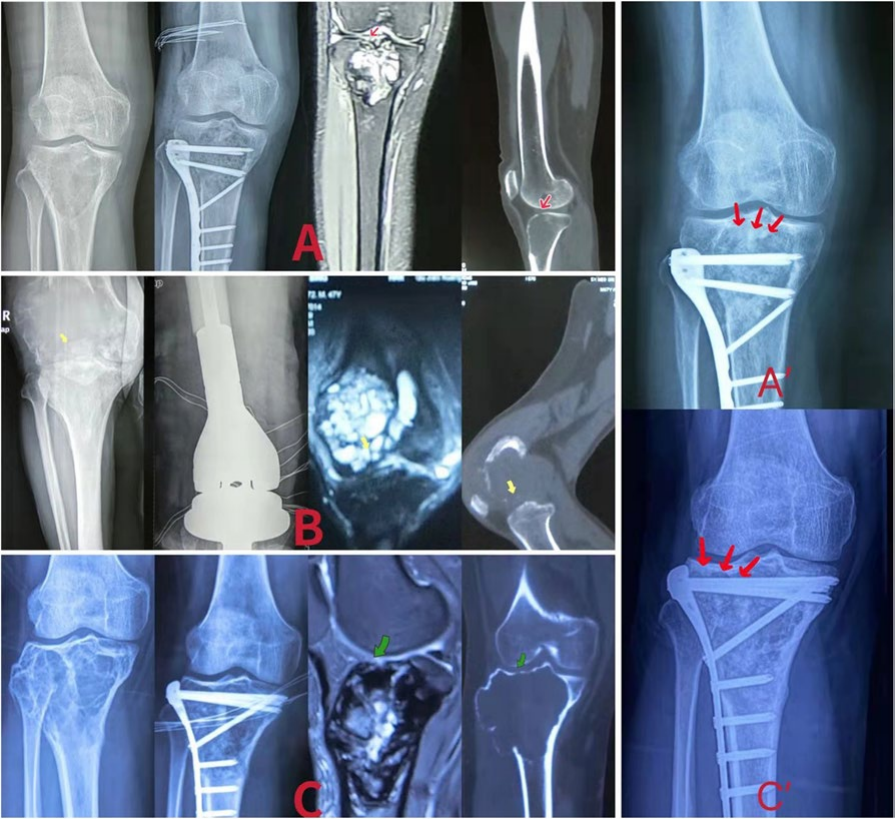
**A** On MRI and CT, we could see that the destruction of the articular surface of patient A was much less than 50% of the total articular surface (red arrow). The destruction of the articular surface was not obvious. In this case, we believed that the postoperative joint function would not be greatly affected. So we chose the surgical procedure, ‘triple clear’ (TC). At 3 years after surgery, we could see that the patient’s joint morphology was basically the same as that before surgery, and no obvious postoperative arthritis was observed. In the areas where there was the destruction of the articular surface before surgery, obvious autogenous bone repair response could be seen (A’). **B** All the articular surface of the femur lateral condyle in the patient B was destroyed. The overall range of the damage exceeded 50% of the total articular surface (yellow arrow). At this point, we believed that segmental resection with prosthesis reconstruction was the only option. **C** We could see that patient C had a fairly large lesion, which even broke through the interosseous compartment. It was graded as Companacci III. However, the destruction of the articular surface was not more than 50% of the overall articular surface (green arrow). In this case, we also chose the TC. At 2 years after surgery, we could see that the patient’s joint morphology was consistent with that before surgery, and no obvious manifestations of arthritis or joint collapse were observed. The subchondral bone repaired obviously. The bone density of the residual cavity also increased significantly (C’)

This retrospective study was approved by the Ethics Committee of the Second Hospital of Shanxi Medical University ((2021)YX No.153), and the requirement for informed consent was waived. From 2012 to 2020, 71 patients with GCTB who were treated at our institute were enrolled following the Declaration of Helsinki 1964 (Supplementary table 1). All patients underwent surgeries by the same surgeon. The inclusion criteria were as follows: (1) patients with Campanacci grade II or III GCTB, (2) patients with single-site primary lesions, (3) patients who received segmental resection with prosthesis reconstruction(SR) or ‘triple clear’ surgery (TC), (4) patients with a follow-up time of more than 24 m, (5) patients with consistent results in the preoperative and postoperative pathological examinations, (6) patients without suspicious metastases and (7) SR and TC were both appropriate for every patient’s tumor status. The exclusion criteria were only patients with recurrent lesions. The lesions of 4 patients (2 in the TC group and 2 in the SR group) were located in the proximal humerus, while the others were all located around the knee joint. Of the 71 patients, 30 underwent SR, while 41 underwent curettage combined with adjuvant therapy (TC). Preoperative pathological diagnosis was confirmed by needle puncture. X-ray, computed tomography (CT) and magnetic resonance imaging (MRI) were routinely performed before surgery (Fig. 1). Pulmonary CT was performed to determine the presence of pulmonary metastasis.

### Surgical technique

#### ‘Triple clear’ surgery

**Fig. 2:**
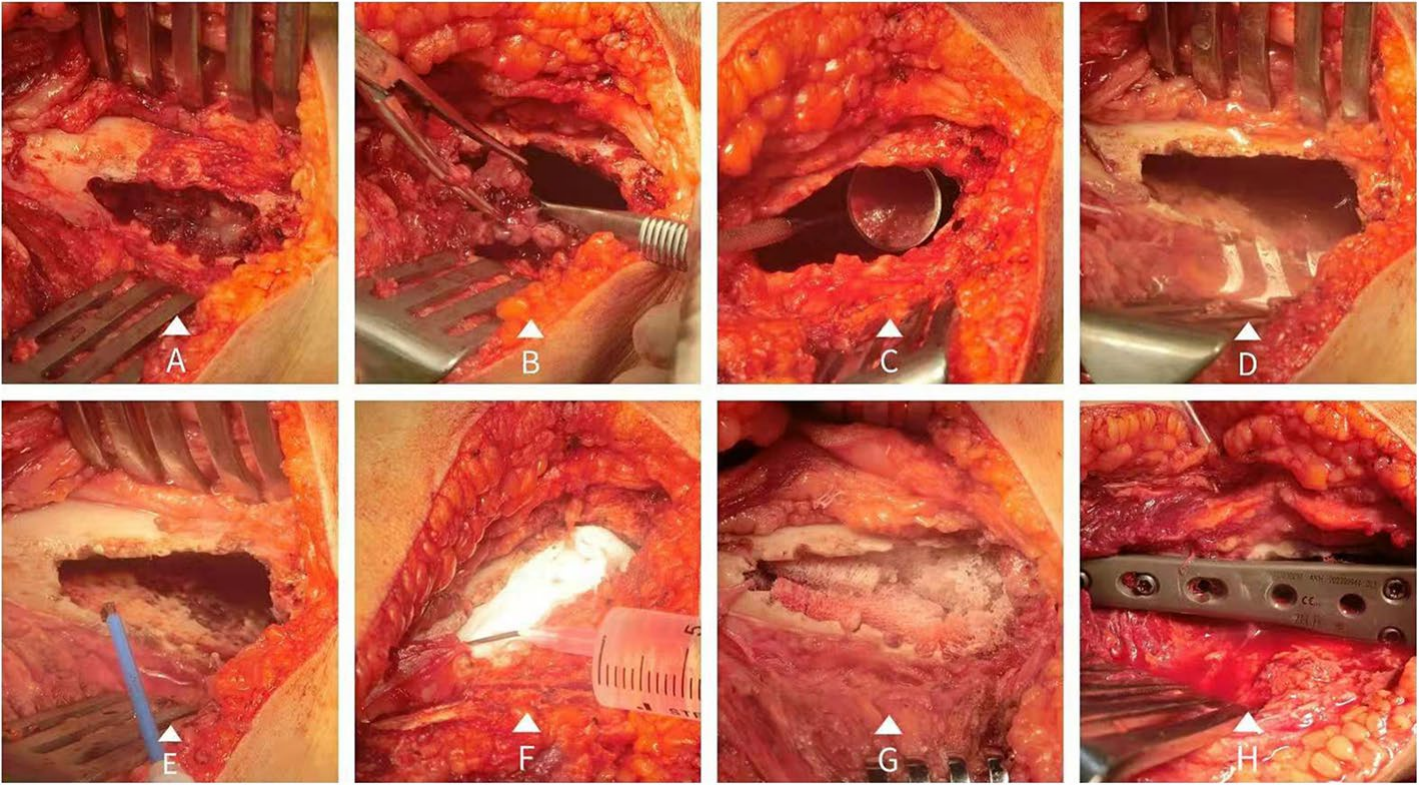
The procedure of TC. **A** A bone window of appropriate size was opened in the area of the lesion. **B** The tumor tissues were scraped out and the residual cavity wall was polished with different types of scrapers in order. **C** Dental endoscopy was used to check the residual cavity and the visual-dead corners. **D** The residual cavity was soaked with anhydrous ethanol for 15 min. **E** An electric knife was used to cauterize the wall of the residual cavity inch by inch. **F** In the final rinse, we used gauze to fill the residual cavity and then injected water, so as to better take out the carbonized necrotic tissues on the wall. **G** Allogeneic bones were compactly inserted into the cavity. **H** The bone window was reset with plates and screws

After anesthesia, an incision was made to expose the lesion thoroughly. The soft tissues around the lesion were strictly protected. A large bone window was made (Fig. 2A). (1) Scrape-out: The macroscopic tumor tissues were removed with a large curette, and the wall of the residual cavity was initially scraped. The wall was not smooth and contained many crests. Middle- and small-sized curettes were successively used to repeatedly scrape the wall inch by inch (Fig. 2B). The whole process lasted at least 20 min. In this process, a dental endoscope was applied to allow for clear observation of the cavity wall (Fig. 2C). The use of the endoscope allowed us to be sure that any operation we performed in the residual cavity was under direct vision. A high-pressure flushing gun and normal saline were then used to wash the residual cavity. Subsequently, AE was used to infuse the residual cavity for 15 min (Fig. 2D). For the residual cavities with pathological fractures or close to the joint surface, AE was smeared on the wall. AE was aspirated with an aspirator. The residual cavity was rinsed again with normal saline. (2) Burn-out: With the assistance of the endoscope, the wall of the residual cavity and invaded soft tissues were cauterized inch by inch (Fig. 2E). The electrotome was used in electrocoagulation mode, and the power was 60 W. The temperature was estimated to reach a range of 150 to 200 ℃. This temperature is far beyond the minimum temperature (50 ℃ [25]) that can cause necrosis of tissues. The cauterization time at each point was 1–2 s. With AE infusion and cauterization, it was believed that deeper inactivation could be achieved than AE or electrocauterization alone. For the wall close to the articular surface, the cauterization power was not changed, but the cauterization time was reduced. Because the cortex of the articular surface is thin, excessive temperatures and long heating times are likely to cause damage to chondrocytes. This damage is often irreversible, which will lead to serious joint complications. After the burnout procedure, the residual cavity was rinsed a third time (Fig. 2F). (3) Flush-out: A flushing gun and normal saline were used to flush the residual cavity several times during the operation. The first wash was to wash off and remove the loose tissues on the surface of the scraped wall. The second wash was to dilute the remaining anhydrous ethanol in the residual cavity, which avoided the combustion of AE during electrocauterization. The third irrigation was to remove the carbonized tissues and reduce the subsequent inflammatory response after electrocauterization. The frequency of each irrigation depended on the different conditions. Finally, the residual cavity was fully filled with bone cement (PALACOS®R + G) or allografts (Fig. 2G). The bone window was closed, and fixation was performed with plates and screws (Fig. 2H). After the drainage tube was placed, the tissues were sutured layer by layer.

#### Segmental resection with prosthesis reconstruction

The procedure is briefly described below. After anesthesia, the patient was placed in an appropriate position. A tourniquet was applied to the proximal end of the affected limb. The tourniquet was inflated after one minute of elevation without displacing blood. For a giant cell tumor of the bone around the knee joint, the incision was made on the medial side. The injury to the nerves and blood vessels should be avoided when exposing the bone with tumor. Osteotomy was performed 5 cm above the tumor boundary. A frozen margin examination was performed for tissues in the medullary cavity of the osteotomy surface. After it was confirmed that no tumor tissue remained at the boundary, a large amount of distilled water and normal saline was used to wash the wound. Then, relevant surgical personnel changed surgical instruments and clothes. New-sterilized surgical sheets were laid around the surgical area. Osteotomy was then performed on the healthy side of the joint. The prosthesis was tried to install into the medullary cavity. The force line of the affected limb and the stability of the prosthesis were checked. After checking, the medullary cavity was filled with bone cement and the tumor prosthesis was installed. The muscles around the prosthesis were sutured and the drainage tube was indwelled. Finally, the subcutaneous tissue and skin were sutured. The joint was moved passively again to see if there was any abnormal movement, abnormal noise or instability. At this point, the operation was completed.

#### Postoperative management and follow-up

The patients in both groups received anti-infection and nutritional support treatment after the operation. When the local drainage volume was less than 10 mL, the drainage tube was removed. And patients were transferred to the rehabilitation department for rehabilitation. The rehabilitation process varied according to the surgery. Patients who received TC only needed absolute immobilization for 3 days. After 3 days, they were able to carry out some mild muscular activities in double crutches. Weight training began when patients were able to raise the leg in a straight position. Patients were recommended to walk alone after 3 weeks and return to normal walking after 1 month.

However, for patients with partial articular surface invasion, we believe that the absolute braking period after TC should be extended to 1–2 weeks. And independent walking should start about 2 months after surgery. For patients who received SR, the rehabilitation process was often painful and lengthy. For patients whose lesions were located at the proximal humerus, absolute immobilization of the upper limb was required for 1 w. For patients whose lesions were located at the distalfemur, 2 weeks of absolute immobilization was recommended [26]. For patients whose lesions were located at the proximal tibia, the period of absolute immobilization was 4 weeks. After the absolute immobilization period, patients could carry out muscular activities and passive rehabilitation training. At 6 weeks postoperatively, patients were encouraged to undergo progressive weight training. Most patients returned to approximately normal levels at 3 months. After patients were discharged from the hospital, the routine follow-up procedure began, including (1) radiography every 3 months to 2 years followed by biannual radiographs for 4 years and annual radiographs in the following years to evaluate oncological prognosis, (2) pulmonary CT biannually to monitor metastasis for 5 years and annually in the following years, (3) functional evaluation by the Musculoskeletal Tumour Society (MSTS) scoring system [27], and (4) evaluation of complications.

### Observation indexes

Various perioperative clinical indicators, including the therapy modality, operation time, Campanacci grade and filling material, were recorded and compared. The pain before and 1 week after surgery of patients in both groups was evaluated by the 0–10 visual analog scale (VAS), with higher scores indicating more serious pain. The limb function of patients in both groups was evaluated by MSTS score at 3 months and 2 years after treatment. Three months after surgery is a transitional time point for the recovery of limb function, while 2 years is a stable point. The maximum score was 30 points, with higher scores indicating better limb function. Follow-up time, recurrence rates, reoperation rates and complication rates were also recorded and compared.

### Statistical analysis

SPSS software version 20.0 (IBM Corp., Armonk, NY) was used for data analysis. Quantitative data are expressed as the mean ± standard deviation, while qualitative data are expressed as the frequency. The chi-square test or Fisher’s exact test was used to compare the rates. The quantitative data were tested for normality before comparison. The data that obey a normal distribution were analyzed using an independent sample *T* test. The data that did not have a normal distribution were analyzed using the rank-sum test. *P* < 0.05 was considered statistically significant. The H–L test can be used to evaluate whether the model maximizes the fit of the model and explains the variance of the model by making full use of the available information. The study can indicate a good model fit superiority effect if *P* > 0.05 and can indicate a poor model construction if *P* < 0.05 (Supplementary table 2). In multi-factor logistic regression, if *P* < 0.05, it means that this variable is significantly different from the dependent variable in the equation.

## Results

### Patient characteristics and surgery

The mean age of patients in the TC group was 33.1 ± 12.6 years. The mean age of patients in the SR group was 38.6 ± 15.4. There was no significant difference in age or sex between the two groups. There were 21 patients with Campanacci grade II and 20 patients with Campanacci grade III in the TC group, of which 4 had pathological fractures. To be more specific, the Campanacci III was further divided into Campanacci III with less than 50% articular surface involvement and Campanacci III with more than 50% articular surface involvement. In the SR group, 9 patients had Campanacci grade II GCTB and 21 patients (15 with less than 50% articular surface involvement and 6 with more than 50% articular surface involvement) had Campanacci grade III GCTB, of which 7 had pathological fractures. Except for patients graded Campanacci III with more than 50% articular surface involvement, there was also no significant difference in the condition of patients between the two groups (*P* > 0.05). The mean operating time for TC was 135.7 ± 38.4 min and that for SR was 174.2 ± 43.0 min. The operating time of TC was significantly shorter than that of SR (*P* < 0.05). Blood loss was less than 150 mL in all patients. For the fillers in the TC group, sixteen patients consented to and underwent bone grafts, while 23 patients with cement filling (Table 2).

**Table 2:** General information

**Variable **	SR* group (***n***=30)	TC* group (***n***=41)	***P*** **-value **
**Mean age, (sd) **	**38.6±15.4**	**33.1±12.6 **	**0.119 **
**Gender, n (%) **			
**M **	**16**	**25 **	**0.520 **
**F **	**14**	**16**	
**Location **			
**femur **	**16 **	**23 **	**0.939 **
**tibia **	**12**	**16**	
**humerus **	**2**	**2**	
**Campanacci grade, n (%) **			
**II**	**9 **	**21 **	**0.074 **
**III **	**21 **	**20 **	
**Campanacci III with < 50% articular surface involvement **	**15 **	**20 **	**0.919 **
**Campanacci III with > 50% articular surface involvement**	**6 **	**0 **	**0.003 (** ***P*** **<0.05) **
**Operating time **	**174.2±43.0 **	**135.7±38.4 **	**0.000 (** ***P*** **<0.05) **
**Duration of follow-up (month) **	**54.7±23.2 **	**59.1±19.5 **	**0.387 **
**Pathological fracture, n(%)**	**7 **	**4 **	**0.219 **

*SR Segmental resection, *TC* Triple clear

**Table 3:** Analysis of observation indexes (except for patients with > 50% articular surface involvement)

**Variable **	**SR group ** **(** ***n*** **=24) **	**TC group ** **(** ***n*** **=41) **	***P*** **-value **
VAS* score			
**pre-operation **	**3.8±1.1 **	**3.3±1.0 **	**0.100 **
**post-operation **	**1.7±0.8 **	**1.6±0.9 **	**0.722 **
MSTS* score			
**three months post-operation **	**18.8±1.3 **	**19.8±1.5 **	**0.010 (** ***P*** **<0.05) **
**two years post-operation **	**24.3±1.4 **	**26.2±1.2 **	**0.000 (** ***P*** **<0.05) **
**recurrence, n (%) **	**2 (8.3%) **	**3(7.3%) **	**0.882 **
**Recurrence, n (before 2017) **	**1(15) **	**2(20) **	**0.727 (P’=0.703) **
**Recurrence rate (before 2017) **	**6.7% **	**10.0% **	
**Recurrence,n (after 2017) **	**1(9) **	**1(21) **	**0.523 (P’’=0.520) **
**Recurrence rate (after 2017) **	**11.1% **	**4.8% **	
**Reoperation, n (%) **	**4 **	**3 **	**0.241 **
**Complications, n (%) **			
**prosthesis loosening **	**2**	**0**	**0.060 **
**infection **	**0**	**1**	**1.000 **
**Postoperative satisfaction **	**24**	**40**	**0.040 (** ***P*** **<0.05) **

*VAS Visual analogue score, *MSTS* Musculoskeletal tumor society

P’: Describe the statistical differences in recurrence rates in the SR group before and after 2017

P’’: Describe the statistical differences in recurrence rates in the TC group before and after 2017

### Prognosis

The follow-up time was 54.7 ± 23.2 months and 59.1 ± 19.5 months, respectively. Three patients (one graded Campanacci II and two graded Campanacci III with less than 50% articular surface involvement) in the TC group developed local recurrences, and all of them underwent a second operation. One patient underwent a second TC surgery and has not developed a second recurrence since then. The other two patients received SR. One patient did not develop recurrence, while the other developed multiple metastases in the lung at 6 months after surgery and eventually died. Three patients (one in the Campanacci III with more than 50% articular surface involvement and two with less than 50% articular surface involvement) in the SR group developed local recurrences. All of them underwent amputations. After the second operation, two patients had no recurrence, while the other died shortly after surgery due to multiple metastases. The recurrence rate (except for patients graded Campanacci III with more than 50% articular surface involvement) was 7.3% in the TC group and 8.3% in the SR group. There was no significant difference between the two groups. Of the eleven patients with pathological fractures, one patient in the SR group developed recurrence, while one patient in the TC group also developed recurrence. There was no significant difference in recurrence rates between the two groups in patients with pathological fractures.

Prior to 2017, 23 patients received SR. There were 9 Campanacci II patients and 14 Campanacci III patients (three with more than 50% articular surface involvement and eleven with less than 50% articular surface involvement). The recurrence rate was 8.7%. Except for the patients graded Campanacci III with more than 50% articular surface involvement, the recurrence rate was 6.7%. Before 2017, 20 patients received TC. There were 14 Campanacci II patients and 6 Campanacci III patients with less than 50% articular surface involvement. The recurrence rate was 10%. There was no significant difference in the recurrence rate between the two groups. However, the indication for TC was significantly broadened after 2017. 21 patients (7 graded Campanacci II and 14 graded Campanacci III with less than 50% articular surface involvement) received TC. The recurrence rate was 4.8% (Table 3). The only recurrent patient was graded Campanacci III with less than 50% articular surface involvement before surgery and recurred in a short period with a rapidly-progressing lesion. Telangiectatic osteosarcomatosis was diagnosed in the examination.

**Table 4:** Grade III tumors with less than 50% involvement

**Variable **	**SR group ** **(** ***n*** **=30) **	**TC group ** **(** ***n*** **=41) **	***P*** **-value **
**Campanacci III with < 50% articular surface involvement **	**15 **	**20 **	**0.919 **
**MSTS score of the patients graded Campanacci III with < 50% articular surface involvement **			
**three months post-operation **	**18.4±2.1 **	**19.9±1.5 **	**0.065 **
**two years post-operation **	**23.4±2.6 **	**26.3±0.6 **	**<0.001 (** ***P*** **<0.05) **
** Recurrence**	**2 (13.3%)**	**2 (10%)**	**0.759**

### Functional recovery and complications

The postoperative VAS scores were significantly reduced in both groups. At 3 months postoperatively, the mean MSTS scores of the TC group and SR group were 19.8 ± 1.5 and 18.8 ± 1.3, respectively. The limb function of patients in the TC group was significantly better than that in the SR group at 3 months after surgery (*P* < 0.05). Moreover, the limb function of patients in the TC group was still better than that in the SR group at two years after surgery (*P* < 0.05). For patients graded Campanacci III with < 50% articular surface involvement, the mean MSTS scores at 3 months post-operation in the SR group and TC group were 18.4 ± 2.1 and 19.9 ± 1.5, respectively (*P* > 0.05). The mean MSTS scores at 2 years post-operation were 23.4 ± 2.6 and 26.3 ± 0.6 (*P* < 0.05)(Table 4). During follow-up, four patients in the SR group presented prosthesis loosening and underwent a second revision surgery. One patient in the TC group presented with a superficial infection in the incision. After sufficient drainage, sterilization and anti-infection treatment, the infection was effectively controlled. During the follow-up, joint stiffness, osteoarthritis and other joint complications were not found. One patient in the TC group was dissatisfied with the procedure, while six patients in the SR group were dissatisfied. There was a significant difference in the degree of satisfaction between the two groups (*P* < 0.05) (Table 3).

The postoperative effects of the two filling materials, bone grafts (BG) and bone cement (BC) were analyzed and compared. The mean MSTS score was 20.0 ± 1.5 in the BG group and 19.6 ± 1.6 in the BC group at 3 months after surgery. At 2 years after surgery, the mean MSTS scores were 26.4 ± 0.6 and 26.4 ± 0.7, respectively. There was no significant difference between the two groups at either time point, which indicated that the two filling materials had no effects on the recovery of limb function. There was also no significant difference in recurrence rates between the two groups. In the BC group, seven patients presented with joint discomfort after surgery, which was statistically significant compared to that in the BG group. The mean age was 27.4 ± 11.0 in the BG group and 37.2 ± 12.6 in the BC group. There was a significant difference in the age between the two groups (*P* < 0.05) (Table 5). A two-factor logistic regression equation was constructed by incorporating age and filler material. The results found that the risk of joint discomfort increased with age and was statistically significant (OR = 1.133, 95% CI 1.011–1.270, *P* = 0.032) (Table 6). Related complications of the adjacent joint were not found during the follow-up.

**Table 5:** Statistics and analysis of data in BG and BC groups

**Variable **	**BG group ** **(** ***n*** **=16)**	**BC group ** **(** ***n*** **=23) **	***P*** **-value **
**Age **	**27.4±11.0 **	**37.2±12.6 **	**0.016 (** ***p*** **<0.05) **
**MSTS score **			
**three months post-operation**	**20.0±1.5 **	**19.6±1.6 **	**0.211 **
**two years post-operation**	**26.4±0.6 **	**26.3±0.7 **	**1.000 **
**Recurrence, n(%)**	**1(6.3%) **	**2(8.7%) **	**1.000 **
**Joint discomfort**	**0**	**7(30.4%) **	**0.029 (** ***p*** **<0.05)**

**Table 6:** Logistic regression about the difference in joint discomfort

**Variable **	**Group **	**b value **	**Wald chi-squared value **	***p*** ** value **	**OR value **	**95％ CI of OR value **
**Age **		**0.125 **	**4.592 **	**0.032 **	**1.133 **	**1.011~1.270 **
**FM **	**BC** ^a^					
	**BG **	**19.948 **	**0.000 **	**0.998 **		

^a^Control group

## Discussion

Since Heijden [18] and other scholars suggested treating GCTB with curettage, burring and adjuvant therapy in 2014, the local recurrence rates have decreased from 30–50% [11, 28, 29, 30, 31, 32] to 6–25% [33, 34, 35]. In this mode of treatment, researchers generally agree that thorough curettage is the first and most important step. Thus, it is necessary to thoroughly open the ‘window’. The size of the ‘window’ usually depends on the size of the lesion on the image. However, this range is often inadequate. The residual cavity after curettage is usually much larger than the size of the window. As a result, there are many ‘blind areas’ that make it impossible to achieve complete curettage. Thus, high-speed burring (HSB) has become a highly praised method by clinicians [36]. However, some researchers with opposing views proposed that the sputtering of particles caused the dissemination of tumor cells [37]. Therefore, it is not plausible that the use of HSB will significantly reduce the recurrence rate in comparison with curettes. In terms of inactivation of the residual cavity, we found that ethanol and electrocoagulation were currently the safest inactivation methods by reading the literature (Table 1). Therefore, we combined these two inactivation methods in the management of residual cavities and described the specific application scheme in detail.

At present, there are also some controversies about the indications of curettage combined with adjuvant therapy. Fraquet [38] believed that segmental resection was the best choice for patients with GCTB, including those with Campanacci grade III GCTB and GCTB located at the distal radius, ulna, fibula, and other nonloadbearing sites. The reason was that Campanacci grade III GCTB was characterized by a large area of bone damage and was bound to the articular surface with varying degrees of damage. However, after the summary of the past practice experience and the improvement and standardization of the surgical procedure, we gradually expanded the indications of curettage. For giant cell tumors of the bone with facet destruction, we think that 50% can be used as a cut-off point. Joint invasion is a cortical breach in the articular surface and articular surface involvement is without breach. For lesions with less than 50% destruction, we believe that TC is feasible. The destruction ratio here refers to the ratio of the affected facet to the total articular surface. Although giant cell tumors of bone often show eccentric growth, most do not cause continuous and complete destruction of the articular surface. On the basis, as long as no more than 50% of the articular surface is destroyed, the self-repair of the subchondral bone and residual cavity accompanied with the support of strong internal fixation can fully guarantee the mechanical support for several years. When the joint surface destruction is more than 50%, curettage and internal fixation are far from providing adequate mechanical support. Due to the large-scale destruction of the subchondral bone, the patients not only need a long period of braking, but also are very likely to have serious arthritic manifestations in a short time and will rapidly progress to the collapse of the articular surface.

On this basis, we try to change the indication of surgical treatment for GCTB. The indications of SR are Campanacci grade III GCTB with pathological fracture, more than 50% joint invasion and/or involvement of most of the metaphyseal. The indications for TC are Camapanacci grade II–III GCTB with or without pathological fracture and slight joint invasion (less than 50% of the articular surface). The damage to the articular surface is evaluated by MRI and CT (Fig. 1). For GCTB patients with a small range of invasion to the articular surface and soft tissues or with pathological fracture, curettage combined with adjuvant therapy is supported with some notable points. The use of chemical reagents can only involve smearing rather than soaking. Soaking may lead to leakage of fluid into the joint cavity and surrounding tissues, which will increase the incidence of complications, such as injury to vessels and nerves. The power and time when used at juxta-articular points should be appropriately reduced to avoid large-scale damage to articular chondrocytes and synovial tissues. And to avoid leakage of bone cement into the joint cavity and surrounding soft tissue, the allogeneic bone is usually chosen as the implant material.

According to the statistical results, we found that the recurrence rates after the two operations were similar, even for GCTB-graded Companacci III and with partial articular surface invasion, which possibly indicated that the effect of tumor cell elimination achieved by TC was similar to that achieved by SR. TC was even used in two patients whose lesions were located at the proximal humerus. And there was no recurrence observed in these two patients. The patients even preserved good function of the upper limbs, which was not achieved by SR. Moreover, for patients with pathological fracture, the recurrence rates after SR and TC were also shown to be similar. The TC group was shown to have a shorter operation time and less blood loss. For the follow-up of limb function, the limb function of patients in the TC group was superior to that of patients in the SR group. The rehabilitation training in the TC group was much easier and the recovery period was shorter, which indicated that the retention of autologous joints had a beneficial influence on postoperative functional recovery. The degree of satisfaction in the TC group was obviously higher than that in the SR group. Even for patients graded Campanacci III with less than 50% articular surface invasion, limb function after TC was shown to be superior to that in SR. For GCTB patients with joint surface invasion, surgeons generally believed that the postoperative function was not ideal and the complications such as articular surface collapse would occur in a short time. However, our clinical experience and research results indicated that for giant cell tumor of the bone with mild articular surface invasion, careful and efficient expanded curettage could not only ensure a low recurrence rate but also would better reserve the limb function. Since patients with GCTB are mostly young and middle-aged, long-term complications caused by artificial joints can be expected. During the follow-up, 3 of 19 patients underwent revision surgeries. The high possibility of revision surgery after SR is an additional financial and emotional burden for patients. At present, except for a superficial infection in one patient, we have not found any operation-related complications in the TC group, which may be due to the insufficient follow-up period.

For a long time, the bone cement has been the best choice for residual cavity filling after curettage of giant cell tumor of the bone, which is because bone cement can perfectly match the osseous voids and provide sufficient mechanical strength. What’s more, it has a tumoricidal ability by thermal polymerization [39]. But this conclusion was based on the specific methods of curettage and different residual cavity management. Gaston [40] analyzed the effect of bone cement on the postoperative recurrence rate after using phenol alone. He concluded that bone cement could significantly reduce the recurrence rate. But this conclusion is obviously one-sided.

After comparing the effects of bone grafts and bone cement, we found that there was possibly no significant difference in the recurrence rate between the BC group and BG group, which might be because the polymerization heat effect of BC was negligible after high-temperature inactivation. Therefore, we think that bone cement filling after thermal inactivation is not helpful to further reduce the postoperative recurrence rate. As for the mechanical support, it was shown that functional recovery was also unaffected by the implant materials. In the BG group, no fracture was found during routine functional exercise after surgery, which was likely to indicate that bone grafting could also fully support the patients’ postoperative functional rehabilitation after strong internal fixation. In addition, we found that some patients in the BC group had discomfort around the joint, which might indicate the occurrence of long-term adverse joint events [41]. We used logistic regression to analyze the relationship between joint discomfort and filling material, age. We concluded, based on our results, that the probability of joint discomfort in postoperative patients may increase with age, while the choice of filler material may not have a significant effect on predicting joint discomfort. Therefore, we believe that it may be possible to predict the probability of joint discomfort by the age of the patient, based on the fact that the patient has identified the filler material. In the BG group, deep infection and transplant rejection associated with bone grafts were not found. On the contrary, we observed an obvious bone repair response in the residual cavity after bone grafts during the follow-up period (Fig. 1A’ and C’). This self-repair and spontaneous fusion will lead to greater comfort and better functional recovery in the long term. In conclusion, allogeneic bone grafts may be a more suitable implant material than bone cement in the long term.

Bone grafting is an important method of treating bone defects. Compared to autologous bone grafts, allogeneic bone grafts are more costly but easily available in terms of quantity and type. The overall infection rate of allogeneic bone grafts ranges from approximately 1.2 to 9% (8% in patients after bone tumor surgery and up to 9% in patients after large allogeneic bone grafts). In the case of benign, degenerative, or traumatic bone defects, infection after allograft bone grafting is rare, in contrast to malignant or aggressive bone tumors where the incidence of postoperative infection is as high as 13%. Approximately 75% of graft infections occur within 4 months of allografting. The availability of allografts is mainly reflected in osteoinduction and osteoinduction. Osteoconduction, also known as scaffolding, relies on osteoinduction to promote healing and replacement of the allograft bone. In contrast, osteoinduction often plays an important role in the early stages of allograft bone healing.

There was one patient in each group who experienced relapse and died due to multiple metastases. At least two rigorous pathological examinations were performed on both patients. The results were consistent. The aim of repetitive pathological examinations is not only to further confirm the diagnosis but also to detect changes in the course of the disease. The rate of metastasis in patients with GCTB is 1.5%. The cellular and molecular biological aspects of this potential invasiveness are unknown. However, once malignant transformation occurs, curettage within the lesion is not recommended. The wrong choice will lead to the artificial spread of tumor cells. Thus, follow-up is a critical aspect of patient management. If large recurrent lesions appear in a short period (less than half a year) and progress rapidly, the possibility of malignant transformation should be considered first. Under these circumstances, SR should be chosen as the treatment option to avoid the worst outcome, even if the images still show the characteristics of benign tumors.

For postoperative rehabilitation, there are many views regarding the mobilization scheme after prosthetic reconstruction. Some surgeons believe that as long as the internal fixation is strong enough, you can move freely on the floor immediately after surgery, especially for a patient receiving a distal femoral prosthesis. Some doctors, on the other hand, consider the early postoperative period to be marked by trauma and painful swelling of the limb and that it is not advisable to move the joints close to the injury site and to wait a few weeks before moving freely for functional exercise. There is no standard general guideline and most decisions are left to the experience and preference of the practitioner.

Although our study was not perfect and part views in the surgical procedure were not entirely innovative, our purpose is definite. We want to convey to clinical researchers and surgeons a careful, detailed and standardized surgical treatment for giant cell tumor of the bone. By the views (full-covered and sequential curettage, endoscopic assistance, standard and combined inactivation, and repeated flushing), we presented a complete surgical procedure to the readers in a comprehensive way, which reflected our rich surgical experience and pursuit to the conscientiousness. We believe that as long as surgeons are rigorous and careful, many patients with GCTB can avoid premature prosthesis replacement and have good postoperative function. The recurrence rate of TC is not significantly low on the whole, which is due to inadequate experience and imperfect surgical procedures in the early stage. From 2017 to 2020, we performed TC on 21 patients (fourteen graded Campanacci III and with less than 50% articular surface involvement). Only one patient had a relapse. This patient was diagnosed with telangiectatic osteosarcoma on biopsy after recurrence. The remaining patients were all tumor-free and had good limb function so far, which fully reflects the importance of refined and standardized surgical treatment. The rigorous surgical spirit is what this article most wants to convey to readers.

## Conclusion

‘Triple clear’ surgery achieved a similar recurrence rate to segmental resection. Patients in the TC group had a shorter operation time, less blood loss and better functional recovery and avoided secondary revision operations, which was of great benefit. Therefore, ‘triple clear’ surgery is preferred for patients with Campanacci grades II–III GCTB and for those with pathological fractures or slight articular surface invasion. For the treatment of recurrent GCTB, the possibility of malignant transformation should also be considered as a factor in the choice of operations. In addition, allogeneic bone grafts may be a more suitable implant material than bone cement in the long term.

## Supplementary Information

**Additional file 1: Table S1.** Demographic and clinical follow-up data of patients. **Table S2.** Hosmer-Lemesho.

## Data Availability

The data used to support the findings of this study are available from the corresponding author upon reasonable request.

## Abbreviations

TC: Triple clear
GCTB: Giant cell tumor of the bone
SR: Segmental resection
AE: Anhydrous ethanol
CT: Computed tomography
MRI: Magnetic resonance imaging
VAS: Visual analog scale
MSTS: Musculoskeletal Tumour Society
BG: Bone grafts
BC: Bone cement
HSB: High-speed burring
