# Supplementary material for: ‘Triple clear’: a systematic and comprehensive surgical process for Campanacci grades II and III giant cell tumors of the bone, with or without pathological fracture and slight joint invasion
Source: World J Surg Oncol. 2023 Mar 29;21:114. doi: 10.1186/s12957-023-02982-2 (PMC10053671; doi:10.1186/s12957-023-02982-2)
Supplement: Supplementary file 1 — Additional file 1: Table S1. Demographic and clinical follow-up data of patients. Table S2. Hosmer-Lemesho. [file 12957_2023_2982_MOESM1_ESM.docx]

**Table 1. Demographic and clinical follow-up data of patients**

N/G A/L TM FM OT(min) FU(m) CG VAS score MSTS score

pre po 3m-po 2y-po

Rec Reo Com

| 1/F1 | 30/F2 | SR | / | 210 | 108 | Ⅲ | 5 | 1 | 20 | 26 | N | Y | PL |
| --- | --- | --- | --- | --- | --- | --- | --- | --- | --- | --- | --- | --- | --- |
| 2/F | 25/T | SR | / | 185 | 104 | Ⅱ | 5 | 2 | 18 | 24 | Y | Y | N |
| 3/F | 49/F | SR | / | 134 | 101 | Ⅲ | 5 | 2 | 18 | D | Y | Y | N |
| 4/M | 49/T | SR | / | 178 | 101 | Ⅲ | 5 | 1 | 19 | 26 | N | N | N |
| 5/F | 18/F | SR | / | 184 | 76 | Ⅲ | 3 | 2 | 14 | 20 | N | N | N |
| 6/M | 51/F | SR | / | 210 | 71 | Ⅲ | 5 | 3 | 20 | 25 | N | N | N |
| 7/M | 33/T | SR | / | 190 | 72 | Ⅲ | 3 | 1 | 20 | 23 | N | Y | PL |
| 8/M | 33/F | SR | / | 140 | 68 | Ⅲ | 3 | 1 | 20 | 26 | N | N | N |
| 9/M | 33/F | SR | / | 165 | 67 | Ⅲ | 2 | 1 | 19 | 24 | N | N | N |
| 10/M | 20/T | SR | / | 300 | 60 | Ⅲ | 3 | 1 | 18 | 24 | N | N | N |
| 11/F | 70/F | SR | / | 175 | 49 | Ⅲ | 3 | 2 | 17 | 22 | N | N | N |
| 12/M | 57/F | SR | / | 170 | 49 | Ⅱ | 5 | 3 | 20 | 24 | N | N | N |
| 13/F | 34/F | SR | / | 125 | 48 | Ⅱ | 3 | 1 | 20 | 24 | N | N | N |
| 14/F | 16/T | SR | / | 125 | 48 | Ⅲ | 3 | 1 | 18 | 26 | N | N | N |
| 15/M | 53/T | SR | / | 195 | 44 | Ⅲ | 3 | 1 | 18 | 24 | N | N | N |
| 16/F | 59/T | SR | / | 150 | 46 | Ⅲ | 5 | 2 | 19 | 25 | N | N | N |
| 17/M | 16/F | SR | / | 115 | 45 | Ⅱ | 3 | 1 | 19 | 25 | N | N | N |
| 18/M | 47/H | SR | / | 195 | 43 | Ⅱ | 5 | 3 | 20 | 22 | N | N | N |
| 19/F | 21/T | SR | / | 180 | 43 | Ⅲ | 3 | 1 | 20 | 25 | N | N | N |
| 20/M | 75/F | SR | / | 160 | 42 | Ⅱ | 3 | 2 | 20 | 26 | N | N | N |
| 21/F | 52/H | SR | / | 120 | 42 | Ⅲ | 5 | 3 | 15 | 20 | Y | Y | N |
| 22/M | 24/T | SR | / | 290 | 39 | Ⅲ | 3 | 1 | 20 | 26 | N | N | N |
| 23/F | 45/F | SR | / | 175 | 39 | Ⅲ | 5 | 1 | 19 | 24 | N | Y | PL |
| 24/M | 33/F | SR | / | 150 | 38 | Ⅲ | 5 | 3 | 20 | 25 | N | N | N |
| 25/M | 27/F | SR | / | 110 | 36 | Ⅱ | 5 | 3 | 17 | 25 | N | N | N |
| 26/F | 41/T | SR | / | 195 | 35 | Ⅱ | 3 | 1 | 18 | 23 | N | N | N |
| 27/M | 35/F | SR | / | 165 | 33 | Ⅲ | 5 | 2 | 19 | 24 | N | N | N |
| 28/M | 32/T | SR | / | 175 | 32 | Ⅱ | 3 | 1 | 19 | 25 | N | N | N |
| 29/F | 43/T | SR | / | 190 | 31 | Ⅲ | 3 | 1 | 20 | 25 | N | N | N |
| 30/F | 37/F | SR | / | 170 | 31 | Ⅲ | 3 | 2 | 18 | 24 | N | N | N |
| 31/F | 37/T | TC | BC | 95 | 104 | Ⅱ | 5 | 2 | 19 | 27 | N | N | N |
| 32/F | 55/F | TC | BG | 115 | 105 | Ⅱ | 3 | 1 | 20 | 27 | N | N | N |
| 33/M | 25/F | TC | BC | 100 | 98 | Ⅲ | 3 | 1 | 20 | D | Y | Y | N |
| 34/M | 26/T | TC | BG | 120 | 91 | Ⅱ | 3 | 1 | 20 | 27 | N | N | N |
| 35/F | 42/T | TC | BC | 99 | 85 | Ⅱ | 2 | 0 | 23 | 26 | N | N | N |
| 36/F | 33/F | TC | BG | 123 | 77 | Ⅱ | 3 | 1 | 21 | 27 | N | N | N |
| 37/F | 16/F | TC | BG | 130 | 76 | Ⅱ | 3 | 1 | 20 | 27 | Y | Y | N |
| 38/M | 29/F | TC | BC | 90 | 74 | Ⅱ | 5 | 3 | 18 | 27 | N | N | N |
| 39/M | 24/T | TC | BC | 133 | 71 | Ⅲ | 3 | 1 | 24 | 27 | N | N | N |
| 40/M | 23/F | TC | BG | 80 | 71 | Ⅱ | 3 | 1 | 24 | 27 | N | N | N |
| 41/F | 19/F | TC | BC | 115 | 70 | Ⅲ | 3 | 1 | 18 | 27 | Y | Y | N |
| 42/M | 28/T | TC | BC | 170 | 70 | Ⅱ | 5 | 3 | 19 | 27 | N | N | N |
| 43/M | 28/T | TC | BC | 170 | 69 | Ⅲ | 3 | 1 | 19 | 25 | N | N | N |
| 44/F | 30/F | TC | BG | 220 | 69 | Ⅱ | 3 | 2 | 18 | 25 | N | N | N |
| 45/F | 28/F | TC | BC | 175 | 66 | Ⅲ | 3 | 2 | 18 | 27 | N | N | N |
| 46/F | 17/F | TC | BG | 110 | 63 | Ⅲ | 3 | 2 | 20 | 27 | N | N | N |
| 47/M | 47/T | TC | BC | 155 | 63 | Ⅲ | 5 | 3 | 20 | 27 | N | N | N |
| 48/M | 33/F | TC | BC | 120 | 62 | Ⅱ | 3 | 2 | 19 | 25 | N | N | N |
| 49/M | 21/F | TC | BG | 150 | 60 | Ⅱ | 5 | 3 | 19 | 27 | N | N | N |
| 50/M | 30/F | TC | BC | 110 | 59 | Ⅱ | 3 | 1 | 19 | 27 | N | N | N |
| 51/M | 23/F | TC | BG | 110 | 56 | Ⅱ | 5 | 3 | 19 | 26 | N | N | N |
| 52/F | 26/F | TC | BC | 140 | 56 | Ⅲ | 5 | 3 | 21 | 27 | N | N | N |
| 53/M | 32/F | TC | BC | 155 | 54 | Ⅲ | 3 | 1 | 21 | 26 | N | N | N |
| 54/M | 38/T | TC | BC | 140 | 54 | Ⅲ | 3 | 2 | 18 | 27 | N | N | N |
| 55/M | 27/F | TC | BG | 55 | 53 | Ⅲ | 5 | 3 | 19 | 26 | N | N | N |
| 56/M | 19/T | TC | BG | 120 | 51 | Ⅱ | 3 | 2 | 18 | 26 | N | N | N |
| 57/F | 38/F | TC | BC | 110 | 48 | Ⅲ | 2 | 1 | 19 | 26 | N | N | N |
| 58/M | 28/T | TC | BG | 130 | 47 | Ⅲ | 3 | 1 | 21 | 26 | N | N | N |
| 59/M | 46/F | TC | BG | 115 | 46 | Ⅲ | 3 | 1 | 21 | 26 | N | N | N |
| 60/M | 42/F | TC | BC | 180 | 44 | Ⅱ | 5 | 3 | 18 | 26 | N | N | N |
| 61/M | 25/T | TC | BG | 170 | 44 | Ⅲ | 1 | 0 | 19 | 26 | N | N | N |
| 62/M | 13/T | TC | BG | 45 | 44 | Ⅱ | 2 | 1 | 21 | 26 | N | N | IN |
| 63/F | 57/F | TC | BC | 150 | 43 | Ⅲ | 3 | 2 | 18 | 26 | N | N | N |
| 64/F | 39/T | TC | BC | 130 | 42 | Ⅱ | 3 | 2 | 19 | 25 | N | N | N |
| 65/M | 64/F | TC | BC | 210 | 39 | Ⅲ | 3 | 1 | 21 | 26 | N | N | N |
| 66/M | 68/T | TC | BC | 200 | 38 | Ⅲ | 3 | 1 | 19 | 26 | N | N | N |
| 67/F | 36/H | TC | BG | 150 | 35 | Ⅲ | 3 | 1 | 20 | 26 | N | N | N |
| 68/M | 45/T | TC | BC | 180 | 33 | Ⅱ | 3 | 1 | 21 | 26 | N | N | N |
| 69/F | 36/T | TC | BC | 170 | 32 | Ⅱ | 3 | 1 | 19 | 26 | N | N | N |
| 70/M | 24/F | TC | BG+BC | 155 | 31 | Ⅱ | 5 | 3 | 20 | 26 | N | N | N |
| 71/F | 41/H | TC | BG+BC | 170 | 31 | Ⅲ | 2 | 0 | 21 | 26 | N | N | N |

N/G:number/gender; A/L:age/location; TM:therapeutic modalities; FM:filling materials; OT(min):operating time; FU(m):follow- up(month); CG:Campanacci grade; VAS:Visual Analogue Score; pre:pre-operation; po:post-operation; MSTS: musculoskeletal tumor society;3m-po: three months post-operation; 2y-po: two years post-operation; Rec:recurrence; Reo:reoperation; Com:complications; F1:female; M:male; F2:femur; T:tibia; H:humerus; SR:segmental resection; TC:triple clear; BC:bone cement; BG:bone graft; PL:prosthesis loosening; IN:infection

Table2.Hosmer-Lemesho

| Step | chi-square | degrees of freedom | p value |
| --- | --- | --- | --- |
| 1 | 2.412 | 8 | 0.966 |
